# Process Evaluation of a Clustered Randomized Control Trial of a Comprehensive Intervention to Reduce the Risk of Cardiovascular Events in Primary Health Care in Rural China

**DOI:** 10.3390/ijerph17114156

**Published:** 2020-06-10

**Authors:** Guanyang Zou, Wei Zhang, Rebecca King, Zhitong Zhang, John Walley, Weiwei Gong, Min Yu, Xiaolin Wei

**Affiliations:** 1 School of Economics and Management, Guangzhou University of Chinese Medicine, Guangzhou 510006, China; zgy1021@hotmail.com; 2 Department of STDs Prevention and Control, Dermatology Hospital, Southern Medical University, Guangzhou 510091, China; krystalwz19@yahoo.com; 3 Nuffield Centre for International Health and Development, University of Leeds, Leeds LS2 9JT, UK; r.king@leeds.ac.uk (R.K.); j.walley@leeds.ac.uk (J.W.); 4 Division of Clinical Epidemiology &Institute of Health Policy, Management and Evaluation, Dalla Lana School of Public Health, University of Toronto, Toronto, ON M5T 3M7, Canada; zhitong.zhang@utoronto.ca; 5 Zhejiang Provincial Centre for Disease Prevention and Control, Hangzhou 310052, China; wwgong@cdc.zj.cn (W.G.); myu@cdc.zj.cn (M.Y.)

**Keywords:** cardiovascular diseases prevention, primary care, process evaluation, China

## Abstract

Background: Cardiovascular disease (CVD) is a major public health challenge in China. This study aims to understand the processes of implementing a comprehensive intervention to reduce CVD events in areas of drug therapy, lifestyle changes, and adherence support in a clustered randomized controlled trial (cRCT). This trial consisted of 67 clusters spanning over 3 years in Zhejiang Province, China. Method: A qualitative process evaluation was nested within the cRCT conducted in 9 township hospitals with 27 healthcare providers, 18 semi-structured interviews, and 23 observational studies of clinical practices within the intervention arm. Results: Effective and repeated trainings using an interactive approach were crucial to improve the prescribing behaviour of family doctors and their patient communication skills. However, the awareness of patients remained limited, thus compromising their use of CVD preventive drugs and adoption of healthy lifestyles. Health system factors further constrained providers’ and patients’ responses to the intervention. Financial barrier was a major concern because of the low coverage of health insurance. Other barriers included limited doctor–patient trust and suboptimal staff motivation. Conclusion: Our study suggests the feasibility of implementing a comprehensive CVD risk reduction strategy in China’s rural primary care facilities. However, health system barriers need to be addressed to ensure the success and sustainability of the intervention.

## 1. Introduction

Non-communicable diseases (NCDs), such as cardiovascular disease (CVD), have increasingly becoming a public health challenge caused by an aging global population [1]. Although primary care aims to deliver preventive interventions, primary healthcare (PHC) systems in low- and middle-income countries (LMICs) have limited capacities to provide essential healthcare services to control NCDs [2,3]. In China, CVD is the leading cause of death. It accounts for 45.01% and 42.61% of total deaths in rural and urban areas in 2015, respectively [4]. The number of CVD cases is on the rise and predicted to increase substantially over the next 10 years [4]. In particular, the mortality of the CVD increased substantially, from 150/100,000 in 1990 to 298.42/100,000 in 2015 in rural areas in China [4]. Despite high incidences and mortality of CVD in China, there is a lack of operational and clinical guidelines designed to prevent CVD events for patients with hypertension or diabetes in the PHC facilities including township hospitals and village clinics.

China has achieved significant progress in strengthening its primary healthcare system. One of most promising achievements is the implementation of Essential Public Health Service Policy (EPHSP). In 2009, PHC facilities started to provide essential public health services for hypertensive and diabetic patients. In many cases, family doctors collaborate with public health doctors, nurses and other PHC staffs to provide comprehensive public health management for hypertensive and diabetic patients. These patients receive regular follow-ups from the team based on their diagnoses and disease severities. In routine practice, family doctors provide basic clinical care for hypertensive and diabetic patients following the national guidelines. However, these family doctors often provide ad-hoc care, prescribe medications based on patients’ requests and have limited medical knowledge in the treatment of hypertension, diabetes, and other CVD-related conditions.

In China, the PHC system faces many challenges. These include increasing numbers of aging population and high prevalence of NCDs, large turnover rate of village healthcare workers (‘village doctors’), ineffective financial subsidies creating lack of incentives for cost reduction and health system performance improvements, inefficient healthcare delivery hindered by insurance policies, low human capacity, poor CVD preventions, and costly preventive treatments. These factors are particularly true in rural areas with less developed health services, low patient incomes, and large populations [5,6,7,8].

To address the need for improved CVD prevention in China, a comprehensive intervention was developed to improve CVD preventative care in patients who were hypertensive with a 10-year CVD risk of >/20% according to the Asian equation, and in patients with high CVD risks from their diabetic conditions [9]. Major interventions comprised of initial and refresher trainings for providers, a clinical and prescribing guide, health education, and adherence support. Patients met with their family doctors on a monthly basis. This could either take place in township hospitals, health stations, village clinics (branches of township hospitals), or family doctor home visits. Family doctors recommended low doses of two anti-hypertensive medications (or only one drug if patient is diabetic), a statin and aspirin (unless contraindicated). In addition, health education regarding smoking cessation, low salt, oil, and alcohol intake were provided to patients during their monthly follow-up appointments. Treatment supporters were selected along with patients to encourage adherence to drugs and lifestyle changes [9]. The care package was designed to be embedded within the essential public health system in China. Following a pilot study to assess the feasibility of these interventions described above [10], a cluster randomized controlled trial (cRCT, consisted of 67 clusters) was conducted over a 3-year timeframe (i.e., December 2013 to March 2017) in Zhejiang Province to evaluate its effectiveness at reducing the incidences of patients with acute CVD conditions. The interim analysis (published elsewhere) suggested that the intervention achieved significant improvements in prescribing practices among family doctors and some lifestyle modifications among patients [11]. Other large CVD preventative studies have reported the effectiveness of the interventions in LMICs [12,13]. However, very few have explored the processes to deliver such interventions. This study, conducted alongside the cRCT, aims to understand the processes of delivering the above interventions and identify factors affecting its delivery in the health systems.

## 2. Materials and Methods

### 2.1. Study Setting

Zhejiang Province is located in the East Coast of China where CVD is the most common cause of death [14]. In 2012, incidence and mortality due to acute CVD events, specifically heart diseases and stroke, were 367.0 and 127.1 per 100,000, respectively. Total case-fatality from CVD events was 34.6% [14]. Since 2009, Zhejiang created a chronic disease surveillance system that covers all counties in the province [14]. Each health facility is obligated to report CVD events to Zhejiang Provincial Centre for Disease Control and Prevention. Township hospitals, the major providers of primary care, are also included in the chronic disease surveillance system [14]. In Zhejiang, the National Rural Health Insurance Scheme only covered 30% of outpatient costs, thus leaving patients paying the remainder 70% out-of-pocket when we conducted this intervention [11].

### 2.2. Data Collection

**Figure 1:**
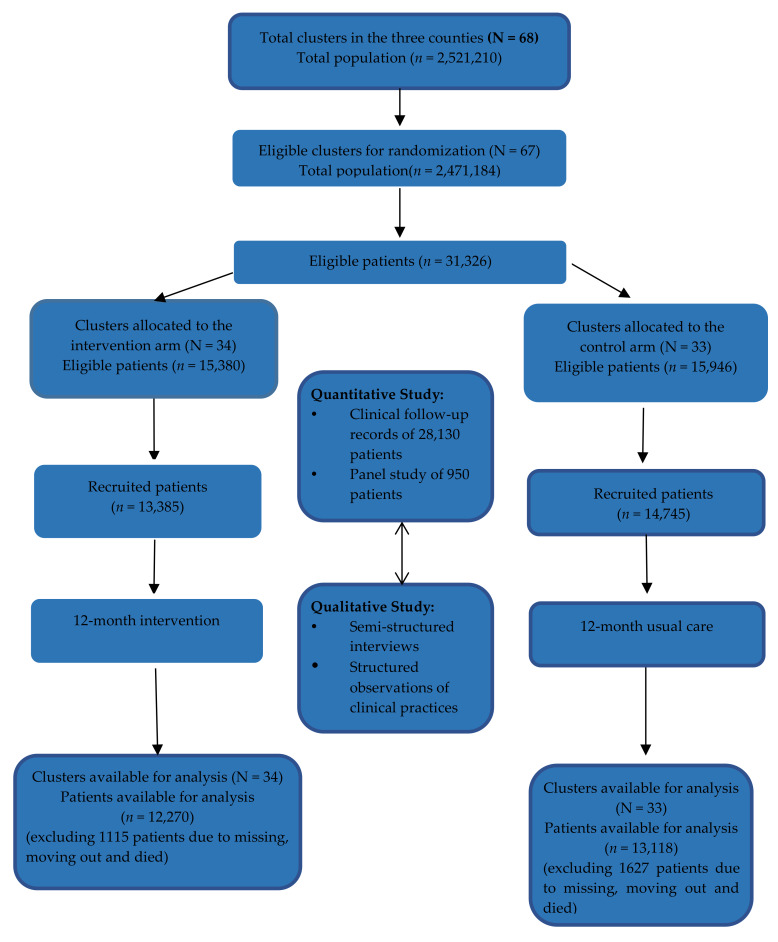
Flow chart of cRCT at 12-month follow up. Note: Adapted from “Implementation of a Comprehensive Intervention for Patients at High Risk of Cardiovascular Disease in Rural China: A Pragmatic Cluster Randomized Controlled Trial.” by Wei et al., 2017, PLoS One 2017, 12 (8), e0183169. doi.org/10.1371/journal.pone.0183169 [11]. Eligible and exclusion criteria of clusters and patients are also detailed in that paper [11].

We nested a mixed-method process evaluation within the cRCT (Figure 1), informed by the Grant et al. framework [15]. In this cRCT, we recruited 13,385 and 14,745 patients in the intervention arm (34 townships) and control arm (33 townships), respectively. Specifically, 48.1% and 49.6% of the patients were male; 98.1% and 98.4% were married; 78% and 75% only attended primary school; the annual household income was 5648 and 6368 USD (1 USD = 6.25 RMB approximately at the time of study); the annual per capita income was 1920 and 2192 USD; 66% and 62% had hypertension and 34% and 38% only had diabetes in the intervention and control arm, respectively [11]. Quantitative and qualitative process data were collected in parallel (quantitative analysis published elsewhere [11]). The quantitative data was collected to understand the prescription and medication rates, lifestyle changes, and adherence to appointments, based on clinical follow-up records of 28,130 participating patients over a 12-month period. Patient surveys were collected at the time of enrolment (baseline), 12 months and 24 months for 950 patients [11]. This paper reported qualitative study results, which were used to gain insights into real-life implementation processes.

We conducted semi-structured interviews with township hospital directors, family and public health doctors, and patients in three counties. An interview topic-guide was used, after initial piloting. In general, the topic guides covered the processes of intervention delivery, such as training of PHC providers, delivery of evidence-based drug therapies, lifestyle modifications, adherence support, and health system issues related to the delivery of these interventions. We purposely selected three township hospitals in each of the three countries, thus nine study sites in total. Providers were selected based on their profession (1 director; 1 family doctor; 1 public health doctor for each township hospital). Two patients (high- and low- adherers) based on their drug adherence patterns were selected from each hospital. In total, we conducted 45 interviews that included 9 directors, 9 family doctors, 9 public health doctors, and 18 patients who participated in the intervention.

Providers’ and patients’ consent was obtained prior to the interview. Semi-structured interviews were conducted by three researchers (2 females, 1 male), who were trained on qualitative research methods between the months of 6 to 12 during the intervention period. Interviews were conducted in Chinese, audio-recorded, and lasted no more than 30 min.

In addition, 23 structured observations of clinical consultations were conducted during our quarterly quality control visits to the 13 township hospitals in the intervention group between the 3-month and 12-month periods. An observational checklist was used to assess the utility of desk guide, prescribing behavior, lifestyle change recommendations, health education and adherence to follow-up appointments. Researchers also took field notes during these visits.

This study was approved by the University of Leeds Research Ethics Committee on 8 October 2012 (reference HSLTLM/12/010) and the Ethics Committee of Zhejiang Provincial Centre for Disease Control and Prevention on 18 June 2012.

### 2.3. Data Analysis

The qualitative interview data were analyzed using the Framework Approach [16]. Each audio-recorded interview was transcribed using Microsoft Word (MS) in Chinese and then imported into NVivo for coding. Researchers read the initial transcripts twice to gain familiarity with the data (Chinese). A coding framework was developed prior to the analysis (in English) based on the intervention components and existing literature and was updated and agreed among the research team per emerging findings. Three researchers participated in the coding process. Two researchers coded data independently for the initial interviews, and a third researcher acted as an adjudicator. Consistency in coding was reached before coding the remainder of transcripts. Data were organized into groups after all transcripts had been coded. Findings from the groups were then interpreted through discussions and validated by back-checking the original transcripts, before major themes were identified. Team collaboration provided ‘analyst triangulation’ to help improve the quality and creditability of research [17]. Descriptive observation data were integrated with the result of interviews in analysis.

## 3. Results

### 3.1. Training of Family Doctors: Interactive Approach Well Received

The significant changes seen in providers’ prescribing behaviors could be attributed to the initial and refresher training sessions. Generally, training sessions focused on prescribing combined CVD preventive medications and their side effects. We observed that lower priority was given to lifestyle modifications during the training sessions. Each 2-h session was presented in PowerPoint slides, accompanied by interactive elements such as group discussions, role-plays, and question-oriented segments with handouts. From our previous experience, an interactive training approach could motivate and engage participants more effectively than the traditional didactic teaching, which is primarily done with trainers giving the talk and participants receiving the information passively. For instance, in a scenario-based group discussion in a patient with poorly-controlled diabetes, we asked the family doctors to discuss appropriate ways of prescribing CVD preventive medicines, before revealing the best practices for this case. All participating doctors confirmed they have benefited from attending these training sessions, since the sessions enhanced their understandings of CVD-related prevention, and also informed them of the most up-to-date prescribing guidelines and practices:

*“First of all, the mindset of our medical staffs have changed particularly regarding the combined use of CVD preventive medicine. Many of us went to training sessions in Shaoxing where we met with experts in this area. The biggest advantage is that we received updates about standardized medication in the preventative care of CVD…”*

(hospital director, male)

This interactive training approach enabled doctors to understand the new guidelines and improve their communication skills with patients:

*“After the training, I polished my communication skills with patients. I feel closer to them, and there’s trust formed between us.”*

(public health doctor, female)

However, some elderly village clinic doctors who are less educated have demonstrated less receptivity to interactive training. We found that they were resistant to new guidelines even after the training and were reluctant to change their practices accordingly.

### 3.2. Processes of Intervention Delivery

#### 3.2.1. Pharmacological Intervention: Discrepancy between Providers and Patients Responses

The interim analysis showed the prescribing rates of recommended two anti-hypertensive drugs, a statin and aspirin (unless contraindicated) were significantly higher in the intervention arm than the control among both hypertensive and patients with diabetes only at the 12-month follow-up [11]. For instance, around 50%, 91%, and 87% of hypertensive patients were prescribed two anti-hypertensives, aspirin and statins in the intervention arm as compared with 20%, 1.7%, and 1.0%, respectively, in the control arm [11]. In this study, we observed that of the 23 consultations, 20 family doctors prescribed medications according to the desk guide, and 18 counseled patients on medication adherence. Doctors were able to discuss the advantages of taking these preventive measures and the risks of non-adherence to patients. However, our interviews suggested that it was challenging for family doctors to change their conventional prescribing patterns, especially those working in the village health stations. While we found significant improvements in prescribing among family doctors in the intervention arm, patients showed relatively low use of the newly-recommended CVD preventative medications [11]. For instance, at 12 months, the drug-taking rates of two anti-hypertensives were at around 24%, 13%, and 7%, respectively [11]. Our study demonstrated that patients preferred to continue their current regimen, typically a co-formulation of Chinese traditional medicine with an old antihypertensive no longer used in Europe. This may be partly due to patients’ outdated medical knowledge, found commonly among the elderly. Also, there was limited awareness of CVD preventive treatment among patients:

*“Doctor prescribed aspirin and a statin, but I don’t take them anymore. My cholesterol is [now] normal, I feel alright, so I stopped.”*

(Patient, male)

#### 3.2.2. Lifestyle Interventions: Communication is Key between Doctors and Patients

Lifestyle education was mainly performed during patient consultations but was also delivered through proactive phone calls and home visits. In 21 of the 23 observed consultations, family doctors provided education on healthy lifestyles to patients. Interviews with family doctors indicated that effective doctor–patient communication was crucial to improving patients’ knowledge in healthy lifestyles. Most family doctors interviewed have said they would focus on the complications of hypertension and diabetes, especially to non-adherent patients. The family doctors would also discuss the social, financial, and medical impact of poorly-chosen lifestyles to patients:

*“I warn patients that he/she will have a stroke if continues to drink or smoke, and his/her children will have to take care of them. That’s a burden to their children …As a result, some patients have cut down their cigarette intake. Some patients used to smoke a pack [20 cigarettes] a day, but now cut down to probably 7 to 8 cigarettes. It reduced, anyhow.”*

(family doctor, male)

Interviews with both doctors and patients suggested the intervention, particularly health education, helped to improve patients’ awareness of CVD prevention, especially within the scope of healthy lifestyle. The intervention was associated with positive lifestyle changes among patients in the intervention arm during Year 1. Smoking rates were significantly reduced (with 4% reduction absolute) in the intervention arm during the first 12 months compared with the control arm (2.5% increase) [11]. Patients in the intervention arm reported drinking less alcohol (32%) as compared to the control arm (15%), although not statistically significant [11]. However, many patients interviewed remained undetermined to lead a meaningful lifestyle change, especially those who are long-term smokers and drinkers. Although other patients understood the harms of continuing unhealthy lifestyles, they said it was difficult to stop for social reasons:

*“Sometimes I felt ‘mei mian zi’ (‘lose face’ by translation, meaning losing dignity) if I don’t do it [attend a dinner banquet]. People stigmatizes me if I refused. I have to save my ‘mian zi’ (‘face’), so I’m obligated to do it even if I didn’t want to.”*

(patient, male)

Many family doctors complained only limited consultation time could be allocated to health education, as it was perceived as time consuming. Our interviews suggested that limited consultation time compromised the quality of primary care because family doctors faced significant challenges in work overload and understaffing in order to deliver essential public health services:

*“The workload in our clinic is unbearable. We have to measure blood pressure, update medical records (both paper and electronic) per EPHS standards. We all do it as multiple part-time jobs! Maybe we didn’t put our hearts into it…but we complained about the shortage of hands as well.”*

(Hospital director, female)

#### 3.2.3. Treatment Supporter: Key to Adherence Support

The rate of monthly follow-ups at the intervention sites ranged from 80% to 88%. Most family doctors reported having followed up on patients averaging 1-3 times a month. We found successful follow-up adherence with 20/23 (87%) of observed consultations. Most patients were willing to visit the township hospitals, but the long distance traveled to township hospitals was commonly reported as a barrier to appointment adherence for patients living in remote areas.

Overall, 93% (11360/12271) of all patients in the intervention arm had a treatment supporter. Oftentimes, the patient’s spouse or an elderly caregiver was chosen to be the treatment supporter. Younger people were rarely selected because they migrate to economically-developed cities for better opportunities. Most patients commented that daily reminders from their treatment supporters were useful in improving their medical adherence and adapting to a healthier lifestyle:

*“If one spouse gets sick, the other usually takes care of him/her. They monitored their medication usage, and they go for a post-dinner walk on a daily basis. They are co-dependent and hope to grow healthy together.”*

(Hospital director, female)

However, some patients reflected that despite constant reminders from treatment supporters, they still found it quite difficult to quit smoking. Our interview with providers and patients attributed this issue to the treatment supporters not having influential positions within the household.

### 3.3. Health Systems: Relationships, Resources, and Motivation

We found that health system issues affected the delivery of our interventions. These included doctor–patient relationships, affordable CVD preventative treatments, and motivation among PHC staffs.

#### 3.3.1. Perceived Poor Doctor–patient Relationship and Trust

Our interviews showed that patients who trusted their family doctors at the township hospitals were more inclined to accept their recommendations on CVD preventive treatments. However, we also found patients who mistrusted their family doctors:

*“I understand what the doctor is trying to say, [after all] I had been to school for few years. What they said is one thing, but for me to trust them is another. Most of the time, I don’t trust doctors.”*

(Patient, male)

Due to their mistrust on family doctors, many patients preferred to visit a county or tertiary hospital, even drug stores where most CVD preventative medications are available.

Tension between family doctors and patients was obvious in the study sites. Our intervention encouraged the prescription of combined CVD preventive medications. However, patients were wary of the recommended drug therapies from family doctors as they suspected their doctors received monetary incentives from pharmaceutical companies by promoting new drugs:

*“I usually don’t trust doctors; I have seen this a lot on TV. Many doctors go to these TV programs, and talk about how these drugs will work. There was a TV program that talked about diabetes, and they are actually selling the drugs. They have to promote drugs eventually…”*

(Patient, male)

This suggests that, in addition to commonly-reported reasons such as primary care’s underdeveloped technical capacity, patients’ perceptions of family doctors are also influenced by the commercialization of health. Against this backdrop, family doctors were particularly concerned about potential medical disputes associated with the side effects of certain CVD preventive drugs:

*“The doctor–patient relation is strained in China. We don’t need a lot, one or two cases [of reported adverse events] is more than enough. Statins have side effects, so does aspirin. If patients don’t trust doctors in the beginning and the prescribed medications cause side effects, they [patients] will hate the doctors even more.”*

(Hospital director, male)

Indeed, some patients reported side effects from taking CVD preventive drugs with aspirin. Side effects often discouraged them from taking the medication for long-term health benefits. As a patient reported:

*“After taking aspirin that time, I sensed a really bad heartburn so I stopped. I just had it once or twice.”*

(Patient, male)

#### 3.3.2. Poor Affordability of Cardiovascular Disease (CVD) Preventive Medicine

Most township hospitals have a unified approach to drug acquisition through the provincial health authorities and bidding platforms. However, the township hospitals often failed to purchase cost-effective essential medicine from the provincial bidding system because the pharmaceutical companies refused to supply certain drugs due to their low profits. As a result, patients were often prescribed more expensive medications with similar efficacies, i.e., statins.

As our intervention embedded within routine primary care practices, patients had to pay their drugs through insurance and/or out-of-pocket bills. Due to the low reimbursement rates from health insurance, patients often have high out-of-pocket payments. In addition, total reimbursement was limited per annum (70 USD to 93 USD per person per annum) and could be used up quickly. On average, patients interviewed spent 16 USD to 160 USD on the CVD preventive drugs per month. While approximately 50% of interviewed patients complained of the high costs of these medications, the other half perceived the medication costs as acceptable and believed the CVD preventive medication was value-for-money. For instance, one patient adhered to the medication despite a high financial endeavor, as he believed the complications from diabetes would be more significant:

*“Taking these drugs cost me a fortune, probably half of my pension. But undoubtedly I still need to take them. Honestly, I would probably be long gone without taking these medicines.”*

(Patient, male)

#### 3.3.3. Poor Motivation of Primary Healthcare Staff

Many family doctors reported their incomes have dropped since the newly implemented performance-based salary system. In the past, their incomes were associated with the services provided for patients and bonuses from township hospitals. However, the new performance-based salary system and the zero-price mark-up policy that no longer allows family doctors to charge patients 15% of the drug costs have ‘demotivated’ family doctors.

## 4. Discussion

With an increasing aging population in China, PHC system will need to undertake increasing responsibilities for CVD prevention and treatment. This study is one of the first studies that addressed the enablers and barriers regarding the delivery of a comprehensive CVD risk reduction strategy of primary care in rural China (Table 1). Our published quantitative study identified positive responses and adherence among family doctors in delivering pharmacologic and lifestyle modifying interventions to high-risk patients [11]. However, the responses and receptiveness of interventions among patients were mixed, given discrepancies between the rates of prescribing and intake of CVD preventive medicine [11]. Although the interventions achieved positive lifestyle changes among patients in the intervention arm, some patients remained undetermined to healthier lifestyles [11].

By providing effective and repeated trainings using an interactive approach, our study suggests that this is the key to refine family doctors’ knowledge, attitudes, behaviors, and communication skills with their patients. However, given the advanced concept of CVD preventive strategy that advocates for low-dose combined preventive drugs, trainings should be tailored to elderly village doctors, who have received limited medical training.

Similar to another study [18], this study suggests patients have limited awareness on chronic disease management. This may contribute to the poor uptake of CVD preventive drugs (low doses of antihypertensives, statin, and aspirin if not contraindicated), inadequate lifestyle changes, and poor follow-up visits. Taking large quantities of medicines, often taking 3 to 4 tablets at once, further hinders medication adherence. China does not have co-formulation of these drugs such as polypill [19]. Patients still preferred their familiar medications including a co-formulation of an old antihypertensive and traditional Chinese medicine.

Consistent with another study [20], our study suggests social factors are deeply embedded in the challenges of lifestyles modifications such as smoking and drinking. Future intervention should strengthen the quality of health education, for instance, to include social elements and relationships in the delivery of education. This depends on the training of healthcare providers to communicate effectively with their patients. Similar to a previous study [21], we found that effective communication acquired from interactive trainings between doctors and patients have increased patients’ awareness of and adherence to treatments. However, this effect may be limited as our published quantitative study showed relatively low use of CVD preventive medicine and suboptimal lifestyle modification amongst the patients with high risk to CVDs [11]. Furthermore, allocating adequate time spent for physician–patient consultations was challenging in the context of increasing public health workloads for family doctors, which compromised the quality of education and preventative patient care [22].

Similar to a previous study [23], our study suggests that using treatment supporters improved patients’ adherence to chronic disease treatments. As population aging becomes more imperative, it is important to engage family members to support chronic diseases management for the elderly. While another study in China shows mistrust of patients towards PHC providers in different ownership models [24], our study suggests that mistrust among patients with high risk to CVDs seek treatment at the higher-level hospitals instead of PHCs. Again, a previous study showed the importance of doctor–patient relationship in improving adherence to asthma treatments in China [25]. Yet our study further identified how doctor–patient relationships could shape the prescribing behavior and facilitate the use of CVD preventive medicine in the primary care practices. We found that patients who trusted their family doctors had a high acceptance rate to the recommendations of their preventative CVD care. Tensions between doctors and patients were apparent in our study observations. This is likely attributed to patient-perceived physicians’ conflicts of interests from pharmaceutical marketing (i.e., open endorsement of pharmaceutical products). Family doctors also tended to restrict the prescription of preventive medications due to concerns of potential medical disputes arising from possible side effects of certain medications. Indeed, a previous study has suggested that primary hospitals in China had the highest possibility of experiencing serious workplace violence [26]. Pursuit of financial incentives at the system-level has led to the eroded patient–physician trust and relationship in China [27,28].

This study suggests financial barriers remained a critical obstacle hindering patients from taking preventive medications. The government’s essential medicine-purchasing system fails to acquire as many generic drugs as possible at a lower price from pharmaceuticals’ influences. In the PHC post-reform period in China, PHC providers prefer stocking and prescribing more expensive medications [29]. While a previous study showed by having medical insurance could positively impact patients’ primary care experiences in China [30], we found that limited reimbursement from rural health insurance further hindered access to care. Health insurance schemes in China mainly aim to address catastrophic illness with very limited cost coverage in preventive care [31].

Although the intervention mainly improves the prescribing behavior of family doctors, their motivation to improve the quality of primary care may be compromised by the implementation of a zero-price mark-up policy and an innovative performance-based payment system that lowers their income. Our trial was one of the first few interventions using comprehensive strategy to reduce the CVD risk at primary care in LMICs, hence, results of this process evaluation are valuable in the potential replication of intervention within similar contexts. A systematic qualitative inquiry within RCTs into aspects of intervention delivery by using multiple methods and triangulated with the published quantitative results, contributes to the improvement of data interpretation and external validity [32]. This study was conducted in the initial intervention period rather than in later follow-ups of this trial. Thus, one must be cautious when applying the results of this study to explain the final outcome of the trial. However, we believe factors identified in this study influence the execution of the interventions. This is especially true in health system issues for explaining the final outcome based on the observation of our continuous follow-ups during the intervention trial period.

## 5. Conclusions

Our study suggests the feasibilities of implementing a comprehensive CVD risk reduction strategy in the rural primary care setting. Improving doctor–patient communication and counseling skills through effective trainings and guidelines are critical to ensure the success of intervention, as this helps to improve patient awareness, medication use, and lifestyle modifications. However, the success and sustainability of these interventions will depend on a need to address certain health system issues, including improving doctor–patient trust and communication, eliminating financial barriers from health insurance reimbursement policies and drug purchasing system, as well as increasing motivation among primary care staffs.

## Figures and Tables

**Table 1:** Enablers and barriers of delivering a comprehensive intervention to reduce the risk of cardiovascular events in primary health care in rural China.

Themes		Enablers	Barriers
**Training of Family Doctors**		Interactive training	Lower priority given to lifestyle modifications; Elderly village clinic doctors less receptivity to interactive training and new guidelines
		motivated and engaged family doctors more effectively than the traditional didactic teaching enhanced their understandings of CVD-related prevention; informed them the most up-to-date prescribing guidelines and practices; helped them to better understand the new guideline and improved communication skills with patients	
**Processes of Intervention Delivery**	Pharmacological Intervention	High provider adherence to prescribed medications; Doctors able to discuss the advantages of taking preventive measures and risks of non-adherence to patients	Challenging for family doctors to change their conventional prescribing patterns, especially those working in the village health stations; Low use of CVD preventative medications among patients as they preferred to continue their current regimen, partly due to their outdated medical knowledge, commonly among elderly, and limited awareness of CVD preventive treatment
	Lifestyle Intervention	Good provider adherence to lifestyle education; Effective education and doctor–patient communication crucial to improving patients’ knowledge and awareness in healthy lifestyles, e.g., discussion of the social, financial, and medical impact of poorly-chosen lifestyles to patients	Patients undetermined to lead a meaningful lifestyle change, especially long-term smokers and drinkers due to social reasons (e.g., concern of losing face); Limited consultation time due to heavy work overload and understaffing, compromising the quality of education
	Follow-up appointment and treatment supporters	Regular follow-up with patients; Most patients had a treatment supporter (usually patient’s spouse or an elderly caregiver); Daily reminders from treatment supporters useful in improving patients’ medical adherence and adapting to a healthier lifestyle	Long distance traveled to township hospitals as a barrier to appointment adherence for patients living in remote areas; Effect of treatment supporters could be limited without having influential positions within the household
**Health Systems**	Doctor–patient relations	Patients who trusted their family doctors more inclined to accept their recommendations on CVD preventive treatments	Mistrust between family doctors and patients not uncommon, due to perceived pharmaceutical and commercial influence and potential medical disputes associated with the side effects of certain CVD preventive drugs
	Affordability of CVD preventive medication	Not identified	Patients were often prescribed more expensive medications with similar efficacies as township hospitals often failed to purchase cost-effective essential medicine; Low reimbursement from health insurance that drugs costs were effectively most out-of-pocket
	Motivation of primary healthcare staff	Not identified	New performance-based salary system and zero-price mark-up policy ‘demotivated’ family doctors
